# Glutathione *S*-transferase (GST) of American Cockroach, *Periplaneta americana*: Classes, Isoforms, and Allergenicity

**DOI:** 10.1038/s41598-017-18759-z

**Published:** 2018-01-11

**Authors:** Nitat Sookrung, Onrapak Reamtong, Rojana Poolphol, Nitaya Indrawattana, Watee Seesuay, Nawannaporn Saelim, Pongsakorn Tantilipikorn, Chaweewan Bunnag, Wanpen Chaicumpa, Anchalee Tungtrongchitr

**Affiliations:** 1 Department of Research and Development, Faculty of Medicine Siriraj Hospital, Mahidol University, Bangkok, 10700 Thailand; 2 Center of Research Excellence on Therapeutic Proteins and Antibody Engineering, Faculty of Medicine Siriraj Hospital, Mahidol University, Bangkok, 10700 Thailand; 3 Department of Molecular Tropical Medicine and Genetics, Faculty of Tropical Medicine, Mahidol University, Bangkok, 10400 Thailand; 4 Graduate Program in Clinical Pathology, Faculty of Medicine Siriraj Hospital, Mahidol University, Bangkok, 10700 Thailand; 5 Department of Parasitology, Mahidol University, Bangkok, 10700 Thailand; 6 Department of Microbiology and Immunology, Faculty of Tropical Medicine, Mahidol University, Bangkok, 10400 Thailand; 7 Department of Oto-rhino-laryngology, Faculty of Medicine Siriraj Hospital, Mahidol University, Bangkok, 10700 Thailand

## Abstract

Insect glutathione *S*-transferases (GSTs) play important roles in insecticide/drug resistance and stress response. Medically, GSTs of house dust mites (*Dermatophagoides pteronyssinus* and *Blomia tropicalis*) and German cockroach (*Blattella germanica*) are human allergens. In this study, classes, isoforms and B-cell and allergenic epitopes of GST of American cockroach, *Periplaneta americana*, the predominant species in the tropics and subtropics were investigated for the first time. Enzymatically active native and recombinant *P*. *americana*-GSTs bound to IgE in sera of all *P*. *americana* allergic patients that were tested. By gel-based proteomics and multiple sequence alignments, the native GST comprises three isoforms of delta and sigma classes. All isoforms interacted with serum IgE of the cockroach allergic subjects. Molecularly, the protein contains six B-cell epitopes; two epitopes located at β1-α1 and β4-α3 regions bound to patients’ serum IgE, indicating that they are allergenic. *P*. *americana* are ubiquitous and their GST can sensitize humans to allergic diseases; thus, the protein should be included in the allergen array for component resolved diagnosis (CRD) of allergic patients, either by skin prick test or specific IgE determination. The GST is suitable also as a target of environmental allergen detection and quantification for intervention of cockroach sensitization and allergic morbidity.

## Introduction

Cockroaches (CR) are ubiquitous. They are pestiferous source of human pathogens and allergens^1^. Although worldwide prevalence of the CR allergy is relatively less than the house dust mite (HDM) allergy^2,3^ clinical manifestations caused by CR are usually more prolonged and severe and often require emergency-room visit, hospitalization and/or intensive care^4^. The CR allergens are prevailed in the environment especially in the infested areas^5,6^. In Thailand, the principal CR species causing human allergic sensitization and morbidity is *Periplaneta americana* (American CR)^7^. Currently, officially recognized *P*. *americana* allergens include Per a 1 (a protein that exists in multiple variants containing different numbers of repeated amino acid domains) which elicit 93% skin reactivity among CR allergic patients and bound to IgE in all patients’ sera^8–10^; Per a 2 or aspartic protease-like protein (42 kDa) bound to IgE in sera of 63% of allergic rhinitis or asthma and rhinitis patients^11^; Per a 3 (Cr-PI), an insect hemolymph or insect storage protein related to arylphorin which caused skin reaction in 93% of CR allergic patients^12^; Per a 6 or troponin C, a 17 kDa protein that bound to IgE in sera of 54% of atopic patients^13,14^; Per a 7 or tropomyosin (a 37 kDa protein with high homology to other invertebrate and vertebrate tropomyosins) which bound to IgE in sera of 41% of atopic and 57% of CR allergic patients^15–17^; Per a 9 or arginine kinase which is a 43 kDa pan-insect protein that reacted to IgE of all CR allergic patients tested^18^; native troponin T (47 kDa) bound to IgE in sera of 17% of *P*. *amaricana* allergic patients^19^; Per a 10 or serine protease, a 28 kDa protein that elicited skin reactivity in 82% of CR allergic patients^20^; and Per a 11 (alpha-amylase; 55 kDa) and Per a 12 (chitinase; 45 kDa) derived from midgut of *P*. *americana* which have been found to react to sera of 83 and 63.8% of allergic patients in immunoblot analysis^21^.

Glutathione *S*-transferases (GSTs) is a large family of intracellular enzymes of aerobic prokaryotes and eukaryotes that the host organisms use for protection against oxidative stress, degenerative conditions, aging, and cancers and also detoxify endogenous and xenobiotic electrophiles such as drugs, herbicides, insecticides^22,23^. GSTs are classified according to their cellular localizations into three major families, i.e., cytosolic, mitochondrial/peroxisomal, and microsomal GSTs^24^. All insect GSTs are cytosolic and further subdivided into at least six classes including delta, epsilon, omega, sigma, theta, and zeta, based on phylogenetic analysis^23^. Delta and epsilon GSTs are arthropod specific^25^. Each GST subunit is approximately 21–28 kDa and may exist as either homo- or hetero-dimeric form^26^.

Most research on insect GSTs focused on their role in insecticide/drug resistance^23,27^. It was reported that increased levels of GST activity contributed to increase detoxification capacity and resistance of the insects to several insecticides^28–31^. For medical aspect, GST of many organisms have been reported as human allergens including *Dermatophagoides pteronyssinus* (Der p 8)^32,33^, *Bromia tropicalis* (Blo t 8)^34^, *Blattella germanica* or German CR (Bla g 5)^35^, *Alternaria alternata* ^36^, *Ascaris lumbricoides* and *Ascaris suis* (Asc s 1 and Asc s 3)^34,37^. The *B*. *germanica* GSTs, i.e., sigma BgGSTS1 and delta BgGSTD1 have been reported as potent human allergens^35,38^. Data on allergenicity and several other attributions of *P*. *americana* GSTs are lacking. Therefore in this study, *P*. *americana* GST classes, isoforms, allergenicity, and B cell epitopes were investigated.

## Materials and Methods

### Serum samples

This study was approved by Siriraj Ethical Committee (COA no. SI268/2008), Faculty of Medicine Siriraj Hospital, Mahidol University, Bangkok. All methods were performed in accordance with the relevant guidelines and regulations by International Ethical Guidelines for Health-related Research Involving Humans. Informed consent was obtained from each subject. Serum samples were isolated from clotted blood aliquots collected from 15 patients who visited Allergy Clinic, Department of Oto-Rhino-Laryngology, Siriraj Hospital, Bangkok. All patients were multiply positive by skin prick test (SPT) to crude *P*. *americana* extract and other allergens, but positivity to the *P*. *americana* extract was more pronounced. Serum specific IgE levels to American CR extract were measured by using ImmunoCAP (UniCAP 250, Instrument Pharmacia Diagnostic AB, Uppsala, Sweden). Serum samples of five subjects who were negative by SPT, IgE ImmunoCAP and IgE-binding ELISA to the *P*. *americana* and other extracts served as non-allergic (normal) controls. A pool of sera of 10* P*. *americana* allergic patients was prepared by mixing 0.5 ml of individual samples.

### Preparation of recombinant GST (rGST) of *P*. *Americana*

Adult cockroaches were caught from houses in Bangkok. They were identified entomologically and only the *P*. *americana* were kept frozen at −80 °C until use. Frozen CR was ground to fine pieces in liquid nitrogen and total RNA was isolated from the powder (100 mg) by using TRIzol reagent (Invitrogen, CA, USA). After checking RNA integrity by agarose gel electrophoresis, cDNA was synthesized from the RNA and used as a PCR template for amplification of full-length GST coding sequence (*gst*). The PCR primers were designed from GenBank database (accession number AY792949; UniProt ID Q1M0Y4); forward: 5′-CCG GAT ATC ATG ACC ATC GAC TTC TAC-3′; reverse: 5′-CGA AAG CTT TCA CTT CTT GGC GAG GTT-3′. The PCR reaction mixture was: 1 μl cDNA, 10 μM each of the forward and reverse primers, 2.5 μl of 10× buffer, 3 μl of 25 mM MgCl_2_, 2 μl of 2.5 mM dNTP, 0.2 μl of 5 units/μl DNA polymerase (Fermentas, Lithuania), and 40.3 μl ultrapure distilled water (UDW). The thermal cycles were: initial denaturation at 94 °C, 5 minutes; 30 cycles of 94 °C for 30 seconds, 50 °C for 30 seconds, and 72 °C for 40 seconds; and final extension at 72 °C for 7 minutes. The PCR product was verified by DNA sequencing before cloning into pKRX-T (Gentaur, Belgium), subcloned into pET20b^+^(Novagen, Merck, Germany) expression vector, and put into BL21 (DE3) *E*. *coli*. A transformed *E*. *coli* colony was grown in isopropyl β-D-1-thiogalactopyranoside (IPTG) (Affimetrix, OH, USA) conditioned-Luria-Bertani (LB) broth (Himedia, India). The recombinant protein was purified from the bacterial lysate by using HISTrap FF affinity chromatography (GE Healthcare Lifesciences) and verified by SDS-PAGE and Coomassie Brilliant Blue G-250 (CBB) staining and LC-MS/MS. Nucleotide and deduced amino acid sequences of the *P*. *americana* rGST was subjected to phylogenetic analysis together with GSTs of other closely related insects to determine percent identity and GST class.

### Preparation of native *P*. *americana* GST (nGST)

Five ml of binding buffer (phosphate buffered saline, pH 7.3) were added to dissolve the CR powder (15 mg). The preparation was sonicated (Sartorius LABSONIC^®^ P sonicator, Germany) in ice-bath at 20 kHz, 2 minutes pulse-on, 3 minutes pulse-off for a total of 15 minutes and then centrifuged at 10,000 *g* at 4 °C for 15 minutes. The clear supernatant was collected, filtered through a sterile 0.45 µm filter, and protein content was quantified by Bradford’s method (Bio-Rad, CA, USA) using bovine serum albumin (BSA) standard curve for calibration. The preparation was loaded onto a GSTrap FF affinity column (GE) that had been equilibrated with the binding buffer. The column was washed thoroughly with the binding buffer to eliminate unbound materials. The column-bound protein was eluted in 1 ml-fractions with a total of 10 ml elution buffer [50 mM Tris-HCl, 20 mM reduced glutathione (Affimetrix), pH 8.0, 5 mM DTT] at a flow rate of 1–2 ml/min. Fractions containing the eluted protein were verified by SDS-PAGE and CBB staining, pooled, dialyzed against distilled water, and lyophilized. The protein was verified by LC-MS/MS.

### SDS-PAGE and Western blot analysis (WB)

SDS-PAGE and WB were performed as described previously^17^. A 4% stacking and 12.5% separating polyacrylamide gels were used in the SDS-PAGE which was performed in a Mini-PROTEAN^®^ 3 Cell (Bio-Rad). Separated proteins in the gels were either stained or electro-blotted onto a nitrocellulose membrane (NC) for WB. For WB, empty sites on the blotted NC were blocked with 3% BSA in PBS before placing the membrane in a solution of mouse anti-6× His tag (Abcam, UK). Anti-mouse immunoglobulin-alkaline phosphatase (AP) conjugate (Dako Cytomation, Denmark) and BCIP/NBT substrate (KPL, MD, USA) were used to reveal the 6× His-tagged-rGST band.

### Protein identification by LC-MS/MS

Native and recombinant GSTs were verified by LC-MS/MS as described previously^39^. The generated ion spectra of the peptides from tryptic-digested GSTs were interpreted by using the Turbo SEQUEST algorithm in the BioWorks^TM^ 3.1SR1 software package (Thermo Fisher Scientific) and the nr.fasta database. The protein search parameter was performed as described previously^39^, which included mass tolerance of 1.25 amu, a fragment mass tolerance of ±0.4 amu, methionine (M) oxidation, and threonine (T) or serine (S) phosphorylation. The identified peptides were further evaluated using charge state *versus* cross-correlation numbers (X_corr_). The criteria for a positive identification of the peptides were X_corr_ > 1.5 for singly charged ions, X_cor_ > 2.0 for doubly charged ions, and Xcorr > 2.5 for triply charged ions. A delta correlation (∆Cn) of >0.08 was used as a cut-off for peptide acceptance.

### Determination of enzymatic activity of GST preparations

Enzymatic activities of the nGST and the rGST were determined by using glutathione *S*-transferase assay kit (Sigma-Aldrich., MO, USA) which the 1-chloro-2,4-dinitrobenzene (CDNB) was used as the enzymatic substrate. GST standard (0.25 mg/ml) was provided with the test kit. Active GST catalyzes the conjugation of L-glutathione to CDNB *via* a thiol group of the glutathione. The reaction product, GS-DNB conjugate, could be detected at absorbance 340 nm (A_340nm_). The amount of the product is directly proportional to the GST activity in the sample. In a reaction mixture, 2 µl (0.5 µg) of native, recombinant, and standard GST were mixed individually with 1 ml of substrate solution containing 980 µl Dulbecco’s PBS, 10 µl of 200 mM reduced L-glutathione, and 10 µl of 100 mM CDNB in a 1-ml cuvette. Absorbance 340 nm of the reaction mixture was determined by spectrometer against blank (1 ml non-enzymatic conjugation substrate solution alone) using BioMate^TM^3 series spectrophotometer (Thermo Fisher Scientific). A kinetic program was set for every 30 seconds over a period of 5 minutes after a lag time of 1 minute. Specific catalytic activities of both nGST and rGST (µmol of GST/ml/minute) were calculated.

### Allergenicities of nGST and rGST

Allergenicities (IgE binding frequencies) of the nGST and the rGST were determined by IgE-ELISA. The assay was performed as described previously^17^. The nGST and rGST (5 μg/ml carbonate-bicarbonate buffer, pH 9.6) were added to separate wells (100 μl/well) of a microtiter plate (Costar, MA, USA) and kept at 37 °C until dried. All GST-coated wells were washed and blocked with 200 μl of a blocking solution (1% BSA in PBS) before adding with serial two-fold dilutions of individual sera and the plate was incubated for 3 hours. Wells added with only the serum diluent served as blank. All wells were washed and added with 100 μl of mouse anti-human IgE-biotin conjugate (Southern Biotech, AL, USA; diluted 1:1,000 in PBS-T). Streptavidin-horseradish peroxidase (HRP) conjugate (Dako Cytomation) and ABTS substrate solution (KPL) were used for color development. Absorbance at 405 nm (OD_405nm_) of the content in each well was determined (ELISA reader, Multiscan*EX*, Labsystem, Helsinki, Finland) against the blank. Cut-off OD_405nm_ between positive and negative IgE-ELISA was arbitrarily set at ≥mean OD_405nm_ of non-allergic sera + 2 standard deviations (SD).

### Identification of nGST isoforms and their IgE reactivity

Isoforms of nGST were determined by a gel-based proteomics. Purified nGST was subjected to 2DE as described previously^39^. For the first dimensional electrophoresis, 7 cm-IPG strips and 0.5% pH 3–10 IPG buffer (GE Healthcare) were used. The electrophoresed-IPG strips were then subjected to 12% SDS-PAGE and proteins in the gel were stained by CBB. Gel pieces containing proteins of ~21 kDa were excised from the stained gel and subjected to in-gel tryptic digestion and LC-MS/MS, respectively. Protein orthologues were identified by comparing the peptide sequences of the *P*. *americana*-GST generated from the mass spectrometry with the Arthropoda/Insecta database sequences.

The nGST isoforms were checked for their reactivity to IgE in the pool of CR allergic patients’ sera by 2DE IgE-immunoblotting. The 2DE-separated nGST was electro-transblotted onto an NC, blocked with BSA, and the blot was allowed to react with the CR allergic patients’ serum pool. After keeping at 4 °C overnight, the NC was washed with TBS-T before placing in a solution of appropriately diluted mouse anti-human IgE-biotin conjugate (Southern Biotech) and kept at 25 °C on a rotating platform for 3 hours. Spots of the nGST isoforms bound by the specific serum IgE were revealed by using streptavidin-AP conjugate (Dako Cytomation) and BCIP/NBT substrate (KPL).

### Identification of *P*. *americana*-GST B cell and allergenic epitopes

Linear B-cell epitopes of *P*. *americana*-GST were predicted by using BepiPred 1.0 server. The amino acid sequence of *P*. *americana*-GST was submitted to the server. Three methods available at the server, i.e., BCPred^40^, AAP^41^, and FBCPred^42^ were used for the epitope prediction. Specific threshold and the epitope length were set at 85% and 20–25 residues, respectively. All predicted peptides obtained from individual methods were aligned with the *P*. *americana*-GST sequence to obtain consensus sequences (predicted linear B-cell epitopes). Peptides containing the potential linear B-cell epitopes of *P*. *americana*-GST were synthesized. Moreover, in the case that two of the predicted epitope sequences were close to each other (peptides 2 and 4 of this study), the overlapped peptide between the two sequences was also synthesized (peptide 3 of this study). Binding of the synthetic peptides to antibodies in a pool of *P*. *americana* sensitized human sera (for determining B-cell epitopes) and to IgE in individual allergic patients’ sera (for determining allergenic epitopes) were determined by dot-ELISAs.

### Dot-ELISAs

For detecting binding of the synthetic peptides to antibodies in the pool of the CR allergic patients’ sera, individual synthetic peptides (1 μg) were dotted onto one cm-NC squares. PBS was used as negative peptide control. The NC pieces were blocked with 1% BSA in PBS-T and then placed in the allergic patients’ serum pool. After keeping at 25 °C for 3 hours, the membranes were washed and allowed to react with goat-anti-human IgG-AP conjugate and BCIP/NBT substrate, respectively, with appropriate incubation and washing with PBS-T between the steps. The enzymatic reaction was stopped by rinsing the membranes with distilled water. Color appeared at the peptide-dotted spots indicated that the peptide contained B-cell epitope.

For determining the IgE reactivity of the synthetic peptides, individual peptides were dotted separately onto NC squares (1 μg/dot) and let air-dried. They were blocked with 1% BSA in PBST and probed with individual serum samples (diluted 1:4 in PBST). Serum samples of normal subjects were included in the experiments. After incubating and washing, all NC squares were reacted sequentially with biotin-labeled-goat-anti-human IgE, streptavidin-AP conjugate, and BCIP/NBT substrate, respectively. The peptides that gave the typical colored spots indicated that they contained allergenic (IgE binding) epitopes.

### Locations of the IgE-binding (allergenic) epitopes on the *P*. *americana* GST three dimensional (3D) structural model

Deduced amino acid sequence of the cloned *P*. *americana* GST was submitted to I-TASSER server^43^. The templates used for GST 3D modeling (selected by the server) were PDB ID: 3WYW, 3VK9, 3AY8 and 4PNF. The allergenic peptides were mapped on the 3D modeled structure of the *P*. *americana*-GST.

## Results

### CR allergic patients

Dermographic data of the 15 *P*. *americana* allergic patients and 5 normal subjects as well as clinical diagnosis and results of the skin prick tests and the specific IgE levels among the patients are shown in Table 1.

**Table 1:** Background information of the *P*. *americana* allergic patients and normal controls.

Subject	Sex	Age (years)	Diagnosis	Specific serum IgE to American CR extract (KAU/L)*	Skin prick test was positive to extract(s) of (mean wheal diameter in mm)**
**Patient**					
1	M	56	AR	2.68	American CR (6.1), Dp (4), Mosquito (3.1), House fly (3.1), and Bermuda grass (3.2)
2	F	43	AR	0.72	American CR (6.5), Dp (3.5), Sedge (3.5), Cat (3.3), Sugar cane (3.3), Mosquito (3.2), House fly (3.1), and Kapok (3)
3	F	47	AR	0.8	American CR (5), Dp (4.8), Cat (3.4), Dog (3.3), and Kapok (3)
4	M	21	AR	1.25	American CR (6.1), Dp (4.8), Cat (3.4), and House fly (3)
5	M	32	AR	2.59	American CR (6.4), Dp (5.1), Bermuda grass (4.1), Mosquito (3.5), House fly (3), and Sedge (3)
6	M	34	AR	2.33	American CR (5.1), Dp (5), Cat (3.8), Mosquito (3.2), House fly (3.1), Dog (3), and Sedge (3)
7	F	20	AR	1.71	American CR (4.1), Dp (3.5), Mosquito (3.2), Cat (3.2), Sugar cane,(3.1), and Sedge (3.1)
8	M	28	AR	<0.35	American CR (5.5), Dp (4), Dog (3.9), Cat (3.5), Mosquito (3), House fly (3), and Kapok (3)
9	F	52	AR	0.59	American CR (5.8), Dp (5), Dog (4), Bermuda grass (3.8), Mosquito (3), and House fly (3)
10	F	33	AR	2.81	American CR (6.4), Dp (4.8), Bermuda grass (4), *Cladosporium* spp. (3.1) and Cat (3)
11	M	21	AR	1.9	American CR (7.1), Dp (5), Mosquito (3), Cat (3), House fly (3), and Kapok (3)
12	M	23	AR	2.77	American CR (5), Dp (5), and Df (5)
13	F	29	AR	2.11	American CR (5.5), German CR (5.2), Dp (4.5), and Df (4.3)
14	M	21	AR	1.48	American CR (6), German CR (5.2), Dp (4.8), Df (4.5), Cat (3), and Dog (3)
15	F	22	AR	1.91	American CR (8.1), German CR (6.4), Dp (5.1), and Df (5)
**Control**					
1	F	25	Normal	<0.35	Negative to all allergens tested
2	F	30	Normal	<0.35	Negative to all allergens tested
3	F	35	Normal	<0.35	Negative to all allergens tested
4	M	24	Normal	<0.35	Negative to all allergens tested
5	M	30	Normal	<0.35	Negative to all allergens tested

AR, Allergic rhinitis; CR, Cockroach; C, Control; Df, *Dermatophagoides farinae*; Dp, *Dermatophagoides pteronyssinus*; F, female; KAU/L, Kilo-allergy units/liter; M, male; P, patient.

*Serum IgE levels were measured by using ImmunoCAP (UniCAP 250, Instrument Pharmacia Diagnostic AB, Uppsala, Sweden). Posive level was >0.35 KAU/L.

**Mean wheal diameter (MWD): (the longest wheal diameter + the perpendicular wheal diameter)/2. MWD larger than 3 mm was positive.

### *P*. *americana* nGST and rGST

**Figure 1:**
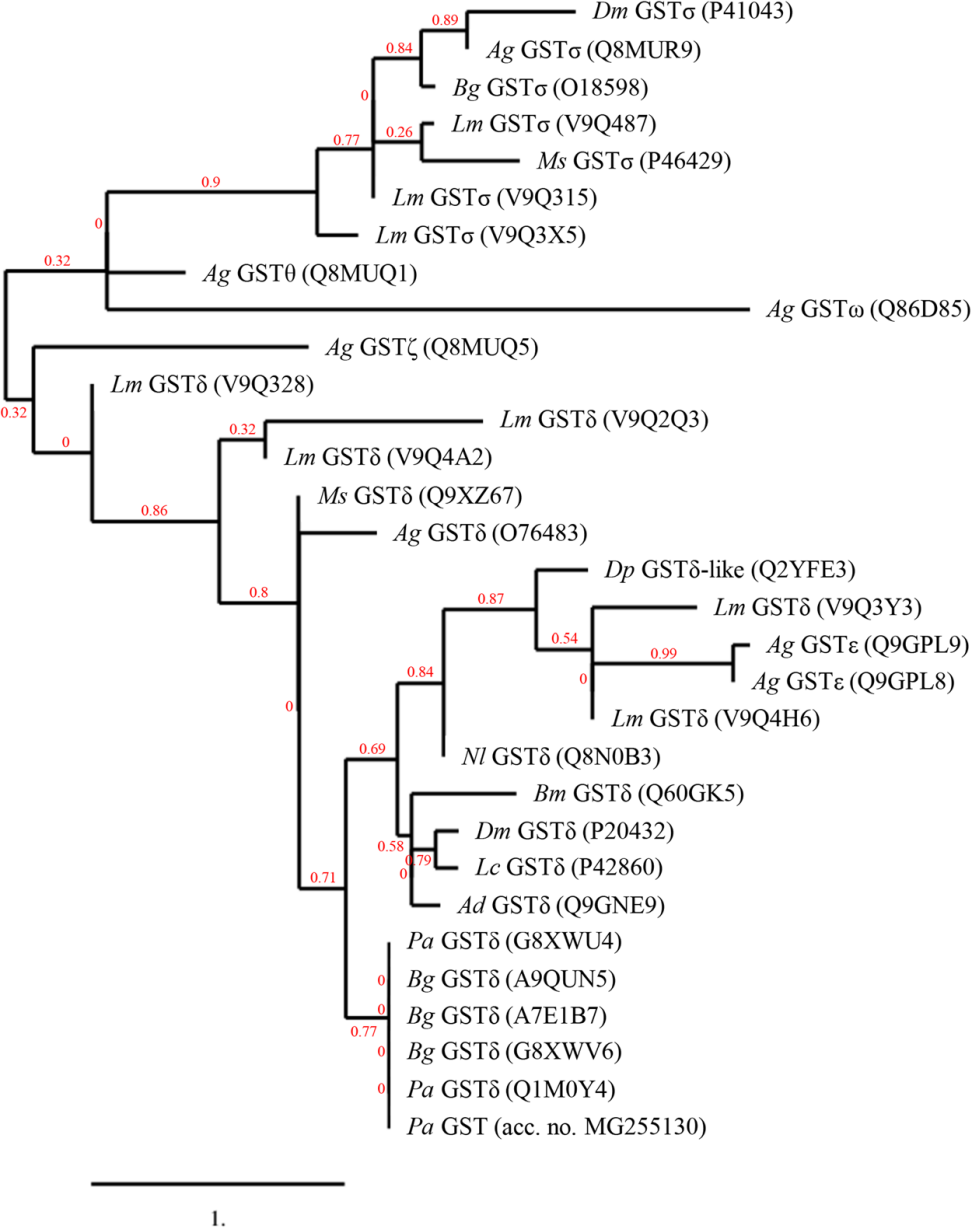
Phylogram of glutathione-S-transferases of *P. americana* and other insects. Ad, *Anopheles dirus*; Ag, *Anopheles gambiae*; Bg, *Blattella germanica*; Bm, *Bombyx mori*; Dm, *Drosophila melanogaster*; Dp, *Dermatophagoides pteronyssinus*; Lc,* Lucilia cuprina*; Lm, *Locusta migratoria*; Ms, *Manduca sexta*; Nl, *Nilaparvata lugens*; Pa, *Periplaneta americana*.

**Figure 2:**
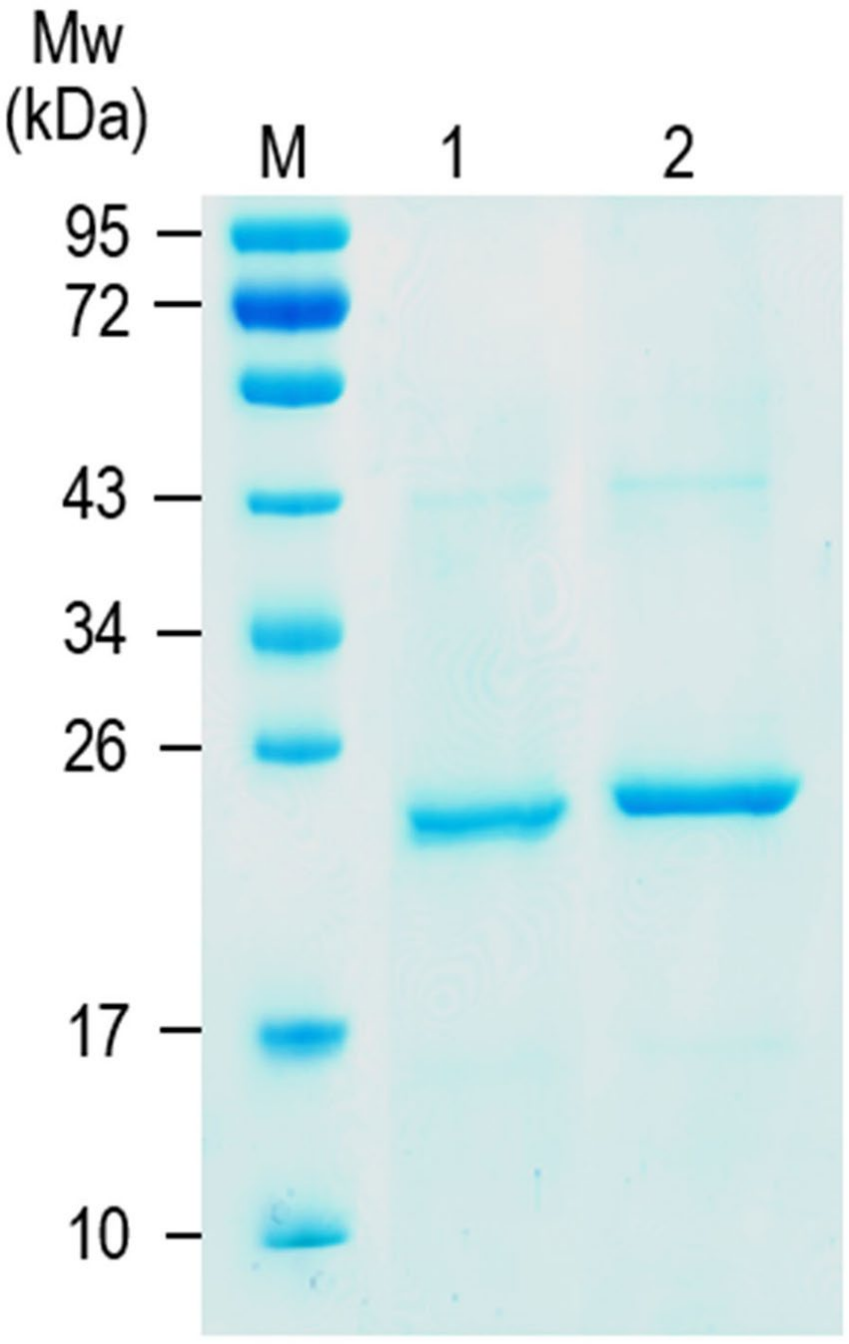
SDS-PAGE-separated patterns of *P*. *americana* native and recombinant GSTs after staining with Coomassie Brilliant Blue G-250 dye. Lane 1, nGST (~23 kDa) eluted from glutathione affinity column; lane 2, rGST (∼25 kDa) purified from *gst*-pET23b^+^-transformed *E*. *coli* lysate M, protein molecular weight (MW) marker. Numbers at the left are relative molecular masses of proteins.

Gene sequence coding for full-length *P*. *americana-*GST (648 bp) was cloned into pKRX-T cloning vector and subcloned into pET20b^+^ protein expression vector. Supplementary Fig. 1 illustrates nucleotide and deduced amino acid sequences of the *P*. *americana*-GST of this study (accession number MG255130). Fig. 1 illustrates a phylogenetic tree of deduced amino acid sequence (216 residues) of the cloned *P*. *americana*-GST and GSTs of homologous and heterologous insects. Alignment (Clustal Omega Multiple Sequence Alignment) and percent identity of amino acid sequence of *P*. *americana*-rGST of this study (accession number MG255130) with GSTs of other insects are shown in Supplementary Fig. 2. The *P*. *americana*-rGST was purified from the *gst*-pET20b^+^-transformed *E*. *coli* (Fig. 2, lane 2) and the protein was verified by the mass spectrometry (Table 2).

**Table 2:** LC-MS/MS Mascot results of peptides generated from in-gel tryptic digestion of *P*. *americana* nGST and rGST after searching against the Swiss-Prot database.

Proteins	Orthologous protein	Accession no.	Number of matched peptides	Protein score	Matched peptide sequence (average peptide score; APS)
nGST	Glutathione *S*-transferase class delta variant 1 (*Periplaneta americana*)	Gi 359326557	5	249	LYFDIGTLYHR (102)
					FGEYYYPIYFAK (52)
					TIDFYYLPGSAPCR (34)
					VTNLMAGEHLTPEFLK (29)
					AILSYLADQYGKDDSLYPK (34)
rGST	Glutathione *S*-transferase class delta variant 1 (*Periplaneta americana*)	Gi 359326557	7	356	AIGVDLNLK (50)
					FKEMCDNLAK (45)
					AILSYLADQYGK (52)
					FGEYYYPIYFAK (64)
					VTNLMAGEHLTPEFLK (48)
					AILSYLADQYGKDDSLYPK (62)
					MNPQHTIPTLNDNGFCLWESR (35)

The nGST prepared from the frozen *P*. *americana* whole body powder and purified by using GST trap column revealed a protein band at ∼23 kDa (Fig. 2, lane 1). LC-MS/MS verified that the protein band was the nGST (Table 2).

### Catalytic activities of nGST and rGST

Enzymatic activities of the nGST and the rGST were 36.65 and 2.93 μmol/ml/min, respectively. The nGST had a much higher enzymatic activity than the rGST.

### Serum IgE reactivities of *P*. *americana* nGST and rGST

**Figure 3:**
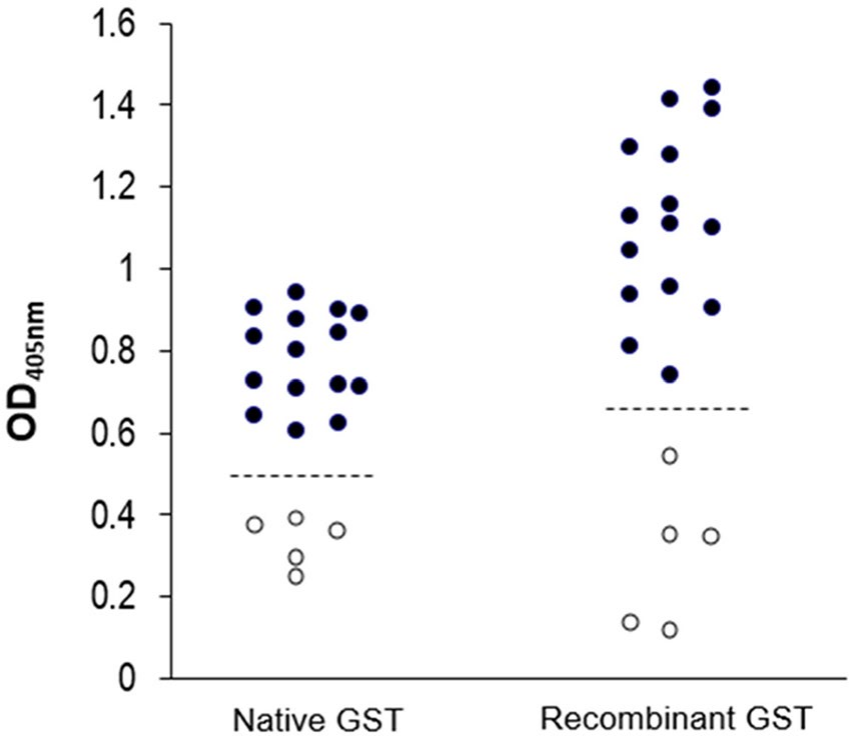
Scattered plots of indirect ELISA OD_405nm_ for determining IgE binding activities (allergenicity) of the *P*. *americana* native and recombinant GST proteins. Both proteins gave significant ELISA signals to all sera of the 15 ACR allergic patients (●) above the 5 normal controls (○). Dotted lines are cut-off OD_405nm_ between the positive and the negative IgE-ELISA which was arbitrarily set at mean + 2 SD of the normal controls.

The mean + 2 SD of IgE-ELISA OD_405nm_ of the non-allergic control sera tested against the nGST and rGST were 0.460 and 0.649, respectively. These values were used arbitrarily as cut-off levels between positive and negative IgE-ELISA and it was found that all of the 15 allergic patients’ sera gave positive IgE-ELISA results to both nGST and rGST proteins (Fig. 3).

### *P*. *americana* nGST isoforms

**Figure 4:**
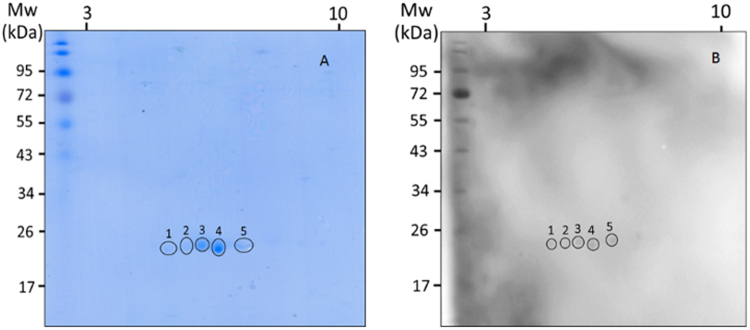
Isoforms of *P*. *americana* nGST and their IgE reactivity. (**A**) Native GST of *P*. *americana* was separated by 2DE at non-linear pH 3–10 and stained by Coomassie Brilliant Blue G-250 dye. Circles indicate protein spots that were subjected to LC-MS/MS for identification. The results revealed that spots 1, 2, and 4 are delta GST while spots 3 and 5 are two different sigma GST variants (see also Table 3). Thus, the *P*. *americana* nGST comprises of three isoforms. (**B**) All GST isoforms reacted to IgE in a serum pool of *P*. *americana* allergic subjects, indicating that they are human allergens.

The 2 DE-pattern of purified *P*. *americana* nGST stained by CBB is shown in Fig. 4A. There are 5 protein spots at ∼21 kDa. The gel pieces containing the 5 protein spots were subjected to LC-MS/MS. The results (Table 3) revealed that tryptic peptides generated from spot nos. 1, 2, and 4 matched with peptides of *P*. *americana* delta variant 1 (accession number Gi 359326557), which the molecular mass was 24606 Da and the pI was 6.44. This protein has possibility of 14 phosphorylation sites and a possible O-glycosylation at residue 116. Peptides of spot no. 3 matched with sigma GST of *Locusta migratoria* (migratory locust) (accession number Gi 565341529) with molecular masses of 23459 Da and pI 6.19. This protein also has 14 possible phosphorylation sites; albeit most sites are different from the delta GST of spots 1, 2, and 4. Peptides of spot no. 5 matched with peptides of *B*. *germanica* sigma GST (accession number Gi 359326585) which has a molecular mass of 23377 Da and pI 6.84 with possible 16 phosporylation sites. Thus, the nGST of *P*. *americana* of this study has three isoforms which belong to delta (one isoform) and sigma classes (two isoforms). By the 2 DE-IgE immunoblotting, all three isoforms of the nGST bound to IgE in a serum pool of the *P*. *americana* allergic patients (Fig. 4B).

**Table 3:** Orthologous proteins of database that contained peptides matched with peptides of proteins in gel plugs 1–5 of 2DE-separated *P*. *americana*-native GST.

Gel plug no.	Access. No.	Protein	Protein score	Mass (Da)	p*I*	No. of peptide	m/z	Ion score	Sequence	Possible phosphorylation sites	Possible O-glycosylation
1	Gi 359326557	GST class delta (*P*. *americana*)	368	24606	6.4	6	424.5294	31	FKEMCDNLAK	11, 42, 56, 72, 78, 83, 85, 100, 105, 114, 145, 166, 176, and 195	116
							466.5781	87	LYFDIGTLYHR		
							780.8726	62	FGEYYYPIYFAK		
							830.3934	65	TIDFYYLPGSAPCR		
							720.6989	44	AILSYLDQYGKDDSLYPK		
							849.3951	79	MNPQHTIPLNDNGFCLWESR		
2	Gi 359326557	GST class delta (*P*. *americana*)	217	24606	6.4	4	780.8727	48	FGEYYYPIYFAK	11, 42, 56, 72, 78, 83, 85, 100, 105, 114, 145, 166, 176, and 195	116
							830.3931	93	TIDFYYLPGSAPCR		
							720.697	55	AILSYLDQYGKDDSLYPK		
							849.3917	20	MNPQHTIPLNDNGFCLWESR		
3	Gi 565341529	GST class sigma (*Locusta migratoria)*	96	23459	6.19	2	434.2514	32	LTYFPVK	5, 8, 24, 42, 46, 65, 97, 105, 110, 114, 151, 163, 176, and 202	N/A
							579.8286	64	YKLTYFPVK		
4	Gi 359326557	GST class delta (*P*. *americana*)	100	24606	6.4	3	780.8705	42	FGEYYYPIYFAK	11, 42, 56, 72, 78, 83, 85, 100, 105, 114, 145, 166, 176, and 195	116
							830.3964	37	TIDFYYLPGSAPCR		
							720.7007	22	AILSYLDQYGKDDSLYPK		
5	Gi 359326585	GST class sigma (*B*. *germanica)*	80	23377	6.84	2	434.2532	31	LTYFPVK	5, 8, 24, 46, 52, 62, 65, 69, 71, 79, 95, 97, 126, 131, 144, and 151	N/A
							486.2729	48	TPVLEIDGK		

### Linear B-cell epitopes and allergenic epitopes of *P*. *americana*-GST

Linear B-cell epitopes of *P*. *americana*-GST predicted by using BepiPred 1.0 server are shown in Supplementary Fig. 3. The BCPred, AAP, and FBCPred methods predicted 2, 3, and 4 epitopic sequences, respectively. All of the predicted peptides were aligned with the *P*. *americana*-GST sequence. They were found to match with five regions (marked in red in the Supplementary Fig. 3) of the GST including β1-α1, α2-β3-β 4, α3-α4, α5-α6, and α8-α9. The consensus peptides of these locations were synthesized (Pep 1, 2, 4, 5, and 6; marked in blue in the Supplementary Fig. 3 and peptides 1, 2, 4, 5, and 6 in Table 4). Because the matched regions α2-β3-β4 and α3-α4 were close to each other, an overlapped peptide of the two regions was also synthesized (Pep 3 in the Supplementary Fig. 3 and peptide 3 in Table 4).

**Table 4:** Sequences of *P*. *americana* synthetic peptides 1–6 which encompassed the 6 predicted *P*. *americana*-GST B-cell epitopes.

*P*. *americana* GST peptide no.	Amino acid sequence and residue numbers	Location on the *P*. *americana*-GST molecule
1	1MTIDFYYLPGSAPCRSVLLA20	β1-α1
2	47MNPQHTIPTLNDNGFCLWESRA68	α2-β3-β4
3	61GFCLWESRAILSYLADQYGK80	β 4-α3
4	72SYLADQYGKDDSLYPKDAKKRALVD96	α3-α4
5	116PIYFAKQAADPEKMKKLEEAFE137	α5-α6
6	187KCKKIVPGYEELNHSGCLKF206	α8-α9

These peptides were used for determining B-cell and allergenic (IgE-binding) epitopes.

**Figure 5:**
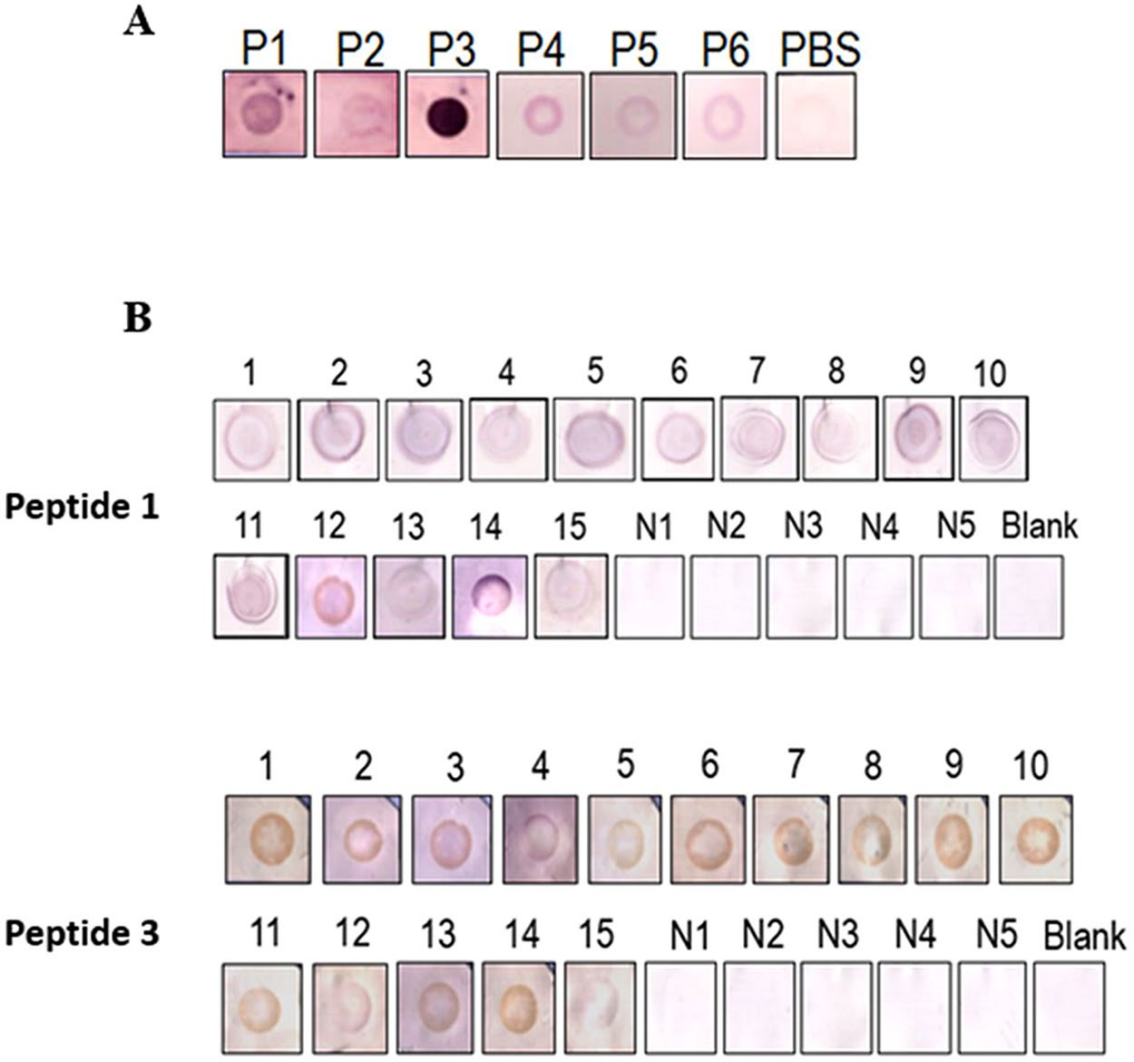
Results of dot-ELISAs for determining binding activities of the synthetic peptides that contained predicted B-cell epitopes of the *P*. *americana*-GST. (**A**) All peptides were bound by antibodies in serum pool of *P*. *americana* sensitized subjects, indicating that the six peptides contain B cell epitopes. PBS was used as a negative antigen control. (**B**) Peptides 1 and 3 were bound by IgE in sera of all 15*P*. *americana* allergic Thai patients (1–15), indicating that these two peptides contained allergenic epitopes. Sera of non-allergic controls (N1–N5) were used as negative IgE control in (**B**). Peptides 2, 4, 5, and 6 were not bound by the serum IgE (data not shown). Blank, peptide probed with PBS.

All synthetic peptides gave positive binding to antibodies in a pool of 10 *P*. *americana* allergic subjects (Fig. 5A), verifying the computerized results that the peptides contained B-cell epitopes. Sera of all *P*. *americana* allergic patients gave positive IgE-dot-ELISA to peptide 1 (1MTIDFYYLPGSAPCRSVLLA20) located between β1 and α1 and peptide 3 (61GFCLWESRAILSYLADQYGK80) located between β4 and α3 of the *P*. *americana*-GST molecule (Fig. 5B) and did not give positive IgE binding with peptides 2, 5, and 6 (data not shown).

**Figure 6:**
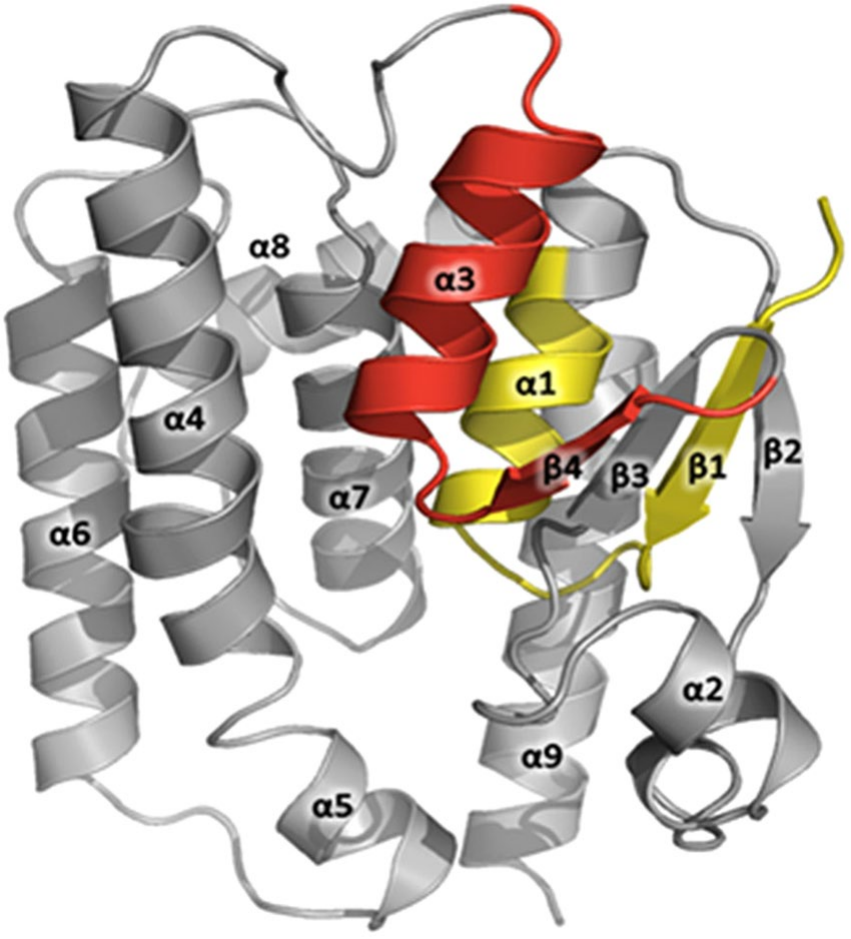
Homology model of the *P*. *americana*-GST three dimensional structure (grey) and locations of peptides 1 (yellow) and 3 (red) that were bound by IgE in sera of the 15 *P*. *americana* allergic patients, implying that these regions contain the GST allergenic epitopes. Peptide 1 is located on β1-α1 while peptide 3 is on β4-α3.

Figure 6 illustrates locations of the two IgE-binding (allergenic) epitopes on the 3D modeled structure of the *P*. *americana*-GST.

## Discussion

American cockroach, *P*. *americana*, is a predominant species and a major source of indoor allergen causing type 1 hypersensitivity in atopic inhabitants of subtropics (such as Taiwan) and tropics (such as Brazil, Malaysia, Singapore, and Thailand), especially in crowded and unhygienic urban cities where their infestation is enhanced^1,44^. Several *P*. *americana*-derived proteins have been recognized as important (major) allergens as they sensitized >50% of cockroach allergic subjects. Glutathione *S*-transferases (GSTs) are indispensable enzymes which insects use to protect themselves against oxidative damage and stress and insecticide toxicity^30^. Most interest on the insect GSTs has been focused on their role in insecticide and drug resistance while other attributions of this protein family seem to be neglected. In this study, three *P*. *americana*-GST isoforms belonging to delta and sigma classes were identified. The roles of the protein in causing human allergy, i.e., allergenicity (specific serum IgE-binding frequency) as well as B-cell epitopes and allergenic epitopes were investigated. To our knowledge, this is the first report on the allergenic attribution of the American cockroach, *P*. *americana*-GST. The protein that was cloned from the GenBank database (accession number AY792949; UniProt ID Q1M0Y4) has been designated Per a 5 (Per a 5.0101) by the IUIS Allergen Nomenclature. The other isoforms reported in this study were based on the tryptic digested peptides generated from the 2DE protein spots that matched with the orthologous proteins of the database. Because the complete amino acid sequences of these isoforms were not available, they were not submitted to the IUIS Allergen Nomenclature.

Enzymatically active native and recombinant *P*. *americana*-GSTs were produced. On the equal weight of both proteins (0.5 μg), the native protein was about 12.5 times more active than the recombinant counterpart. The difference may be because the nGST contains several isoforms belonging to different GST classes whereas the recombinant one is produced from only one cDNA sequence. The multiple isoforms in the nGST might confer additive or synergistic enzymatic activity. Besides, the rGST produced from the transformed BL21 (DE3) *E*. *coli* contains additional 6× His tag (useful for subsequent protein detection and purification) but lacks putative post-translational modifications such as glycosylation and disulfide bridge formation which might impact on the protein folding compared to the native state and hence the less enzymatic activity. After subjecting the nGST and rGST to SDS-PAGE and protein staining, the molecular masses of the native and recombinant proteins were ∼23 and ∼25 kDa, respectively. The larger size of the latter should be from the plasmid franking regions and the 6× His tag. The rGST produced in this study (accession number MG255130) contained 216 amino acids which are similar in residue number to the cloned 24614 Da *B*. *germanica* GSTD1^45^. The *P*. *americana-*rGST has 99.5 and 98.1% sequence identity to the previously reported *P*. *americana*-GSTs (UniProt ID G8XWU4 and Q1M0Y4) and 32.9–82.4% identity to the delta GSTs of other organisms and less so to the other GST classes. Thus, the recombinant *P*. *americana*-GST (accession number MG255130) should belong to the delta class.

Both native and recombinant *P*. *americana* GSTs reacted with IgE in sera of all cockroach allergic patients when tested by indirect ELISA, indicating that the GST is a novel and important (major) allergen of the *P*. *americana*. The ELISA using rGST as antigen gave higher background signal than when the nGST was used. This could be due to the contamination of residual proteins of the *E*. *coli* used as the rGST expression host in the rGST preparation which reacted to the anti-*E*. *coli* that exists naturally in the human sera.

Data on the allergenic repertoire of a protein are useful for understanding the patients’ allergenic response, cross-reacting allergenic determinants among allergens, as well as for properly designing of an engineered therapeutic allergen vaccine/diagnostic material, particularly for personalized immunotherapy and component resolved diagnosis (CRD)^46,47^. Several methods have been used for gaining information on B-cell and allergenic epitopes of an allergen. These include the use of overlapping synthetic peptides or fragments of recombinant allergens^47,48^; mimotope mapping^49^; peptide microarray immunoassay^50,51^; X-ray crystallography and nuclear magnetic resonance techniques^47,52–55^, computerized prediction^56–58^ and specific monoclonal antibody binding and IgE competition assay^58–60^. In this study, a combination of *in silico* methods and synthetic peptide based-immunoassays was used for predicting and determining B-cell and allergenic epitopes of the *P*. *americana-*GST. For the *in silico* prediction of B cell epitopes, three different methods of the BepiPred 1.0 server, i.e., BCPred, AAP, and FBCPred were used. All methods gave a conformed prediction of a peptide located at α2-β3-β4; the AAP and PBCPred methods predicted another peptide at α3-α4; the BCPred and the FBCPred methods predicted another peptide at α5-α6; and only the AAP method predicted two more peptides at β1-α1 and α8-α9. The different results so-obtained suggest that several *in silico* methods should be used for increasing the possibility of finding the potential B-cell epitopes of a particular protein. From the *in silico* prediction, consensus peptides encompassed the potential B-cell epitopes were synthesized and used in the dot-ELISAs for detecting peptides bound by antibodies (B-cell epitopes) and IgE (allergenic epitopes) in sera of *P*. *americana* sensitized subjects. By using the dot-ELISAs, all six synthetic peptides that contained predicted GST B-cell epitopes reacted with antibodies in a serum pool of *P*. *americana* exposed-subjects, implying that the six peptides were part or contained GST B-cell epitopes. Among them were two IgE-binding epitopes located at the β1-α1 and β4-α4 regions of the GST molecule.

In summary, this study provides an insight into characteristics and medically important role of the *P*. *americana-*GST beyond the previously established physiologic roles of the protein in the host defense against toxic substances and stress conditions. Both native and recombinant GSTs of the *P*. *americana* were bound by IgE in sera of cockroach allergic subjects and thus the GST is a novel and major *P*. *americana* allergen. Because cockroaches are ubiquitous, attention should be paid on reducing the insect derived-allergenic proteins from human environment for intervention of allergic sensitization of the naives and clinical aggravation of the sensitized subjects.

## Electronic supplementary material

Supplementary Figures
