# Supplementary material for: Glutathione S-transferase (GST) of American Cockroach, Periplaneta americana: Classes, Isoforms, and Allergenicity
Source: Sci Rep. 2018 Jan 11;8:484. doi: 10.1038/s41598-017-18759-z (PMC5764987; doi:10.1038/s41598-017-18759-z)
Supplement: Supplementary file 1 — Supplementary Figures [file 41598_2017_18759_MOESM1_ESM.pdf]

# Glutathione S-transferase (GST) of American Cockroach, Periplaneta americana: Classes, Isoforms, and Allergenicity

Nitat Sookrung [1,2], Onrapak Reamtong [3], Rojana Poolphol [4,5], Nitaya Indrawattana [6], Watee Seesuay [2], Nawannaporn Saelim [2,5], Pongsakorn Tantilipikorn [7], Chaweewan Bunnag [7], Wanpen Chaicumpa [2,5], Anchalee Tungtrongchitr [2,5,*]

[1]Department of Research and Development, [2]Center of Research Excellence on Therapeutic Proteins and Antibody Engineering, [4] Graduate Program in Clinical Pathology, [5]Department of Parasitology, and [7] Department of Oto-rhino-laryngology, Faculty of Medicine Siriraj Hospital, Mahidol University, Bangkok, 10700, Thailand [3] Department of Molecular Tropical Medicine and Genetics and [6]Department of Microbiology and Immunology, Faculty of Tropical Medicine, Mahidol University, Bangkok 10400, Thailand

```
1    atgaccatcgacttctactacctgcccggcagcgcaccatgccggtcagctctcctggct
      M  T  I  D  F  Y  Y  L  P  G  S  A  P  C  R  S  A  L  L  A    20
61   gccaaggccatcggtgtggatctgaacctcaaagtgacgaacctcatggctggcgaacat
      A  K  A  I  G  V  D  L  N  L  K  V  T  N  L  M  A  G  E  H    40
121  ctcacgcctgagtttcttaagatgaatcctcagcatacgattccagccctaaacaacaac
      L  T  P  E  F  L  K  M  N  P  Q  H  T  I  P  A  L  N  N  N    60
181  gttagctatttgtgggaaagccgagccattctaagtaagctggctaaccggtatggcaag
      V  S  Y  L  W  E  S  R  A  I  L  S  K  L  A  N  R  Y  G  K    80
241  gacgactcgctgtaccgcagtacggcaaaaagcgagctcttgtgggacagagactgtact
      D  D  S  L  Y  R  S  T  A  K  S  E  L  L  W  D  R  D  C  T    100
301  tcgatattgttcccctgtaccattgatacggagaattcttttatgcaatttcatttagca
      S  I  L  F  P  C  T  I  D  T  E  N  S  F  M  Q  F  H  L  A    120
361  aagcaacctgtagatcatgaaaagataaagcaactagagaagcccttcgagtttttgaat
      K  Q  P  V  D  H  E  K  I  K  Q  L  E  K  P  F  E  F  L  N    140
421  aagttcctggaatcgcaacagtttgtggcagaaaataagctcaccattgcggacctggca
      K  F  L  E  S  Q  Q  F  V  A  E  N  K  L  T  I  A  D  L  A    160
481  attgtctcctctgtttccactgctgacatcataggctttgatgtaagcaaatactcaaac
      I  V  S  S  V  S  T  A  D  I  I  G  F  D  V  S  K  Y  S  N    180
541  gtcgccaaatggttcgagaaatgcaagaagattgttccaggctatgaggaactgaatcac
      V  A  K  W  F  E  K  C  K  K  I  V  P  G  Y  E  E  L  N  H    200
601  tccggatgcttgaagttcaaggagatgtgcgataacctcgccaagaagtga
      S  G  C  L  K  F  K  E  M  C  D  N  L  A  K  K  -             216
```

**Supplementary Figure 1. Nucleotide and deduced amino acid sequences of GST of American cockroach, *P. americana*, of this study (accession number MG255130).**

```
GST|Accession no.MG255130   ------------------------------------------------MTIDFYYLPGSA
GSTδ|V9Q2Q3                 -----------------------------------------------MPSIILYGNELSP
GSTδ|V9Q3Y3                 -----------------------------------------------MAPPTLYDNPISP
GSTδ|V9Q4H6                 -----------------------------------------------MSPIILYNHDGSP
GSTδ|V9Q328                 -----------------------------------------------MAGLKLYSVSDSP
GSTδ|V9Q4A2                 -----------------------------------------------MPKLRLYHAEASP
GSTδ|O76483                 ------------------------------------------------MTPVLYYLPPSP
GSTδ|Q9XZ67                 ---------------------------------------------MPSEPIKFYYLAPSP
GSTδ|Q60GK5                 ------------------------------------------------MTIDLYYVPGSA
GSTδ|Q8N0B3                 ------------------------------------------------MPIDLYYVPGSA
GSTδ|A9QUN5                 ------------------------------------------------MTIDFYYLPGSA
GSTδ|A7E1B7                 ------------------------------------------------MTIDFYYLPGSA
GSTδ|G8XWV6                 ------------------------------------------------MTIDFYYLPGSA
GSTδ|Q1M0Y4                 ------------------------------------------------MTIDFYYLPGSA
GSTδ|G8XWU4                 ------------------------------------------------MTIDFYYLPGSA
GSTδ|Q9GNE9                 --------------------------------------------------MDFYYLPGSA
GSTδ|P20432                 -------------------------------------------------MVDFYYLPGSS
GSTδ|P42860                 --------------------------------------------------MDFYYLPGSA
GSTδ-like|Q2YFE3            -----------------------------------------------MSKPIFYYHPFSG
GSTε|Q9GPL9                 -----------------------------------------------MPKPVLYTVHLSP
GSTε|Q9GPL8                 -----------------------------------------------MSNLVLYTLHLSP
GSTσ|P46429                 -----------------------------------------------MPKVVFHYFGAKG
GSTσ|V9Q3X5                 ----------------------------------------------MAPKYKLLYFPFRV
GSTσ|P41043                 MADEAQAPPAEGAPPAEGEAPPPAEGAEGAVEG-GEAAPPAEPAEPIKHSYTLFYFNVKA
GSTσ|Q8MUR9                 -------------------------------------------------------FNVKA
GSTσ|O18598                 ----------------------------------------------MAPSYKLTYCPVKA
GSTσ|V9Q487                 ----------------------------------------------MAAKYKLTYFPIMA
GSTσ|V9Q315                 ----------------------------------------------MAPKYKLTYFPVKA
GSTω|Q86D85                 -----------------------------MSNGKHLAKGSSPPSLPDDGKLRLYSMRFCP
GSTθ|Q8MUQ1                 ----------------------------------------------MSKNLKYYYDLMSQ
GSTζ|Q8MUQ5                 ---------------------------------------MANVDILPESQPILYSYWRSS


GST|Accession no.MG255130   PCRSVLLAAKAIGVDLNLKVTNLM--AGEHLTPEFLKMNPQHTIPTLND---NGFCLWES
GSTδ|V9Q2Q3                 PSRAAKMIAEKLGVDVDFKRTYPI--KGECKKPEYLKINPMHTIPTIID---GPFTLSDS
GSTδ|V9Q3Y3                 PCRLVRLVAGVIGVDLKVVNVKDA--GIDMKSPEMLKKNPQHTVPTLED---NGLCLAES
GSTδ|V9Q4H6                 PCCLVRLVAGVLGLELKKVDISDI--ENGMRNPEMLKKNPQHTIPTIED---DGLVITES
GSTδ|V9Q328                 PSTAVKMALEALALEYTNVEVDFA--AGEHLSDDFSKKNPQREIPCLDD---NGFFLSES
GSTδ|V9Q4A2                 PCRTVRMVARTLGLELQLVDVDLL--QNEHLKPQFLQMNPQHQVPLLDD---NGFYLPES
GSTδ|O76483                 PCRSVLLLAKMIGVELELKALNVM--EGEQLKPDFVELNPQHCIPTLDD---HGLVLWES
GSTδ|Q9XZ67                 PCRAVMMAARALDLELDLIPTNIM--DSDHKTPEYLKMNPQHTIPTMDD---SGFILWES
GSTδ|Q60GK5                 PCRAVLLTAKALNLNLNLKLVDLH--HGEQLKPEYLKLNPQHTVPTLVD---DGLSIWES
GSTδ|Q8N0B3                 PCRNVLLAAKAVGVDLNLKLTDLK--SGQHLTPEFIKLNPQHNVPTLDD---NGFVLNES
GSTδ|A9QUN5                 PCRSVLLAAKAFGVDLNLKVTNLM--AGEHLTPEFLKMNPQHTIPTLND---NGFCLWES
GSTδ|A7E1B7                 PCRSVLLAAKAFGVDLNLKVTNLM--AGEHLTPEFLKMNPQHTIPTLND---NGFCLWES
GSTδ|G8XWV6                 PCRSVLLAAKAFGVDLNLKVTNLM--AGEHLTPEFLKMNPQHTIPTLND---NGFCLWES
GSTδ|Q1M0Y4                 PCRSVLLAAKAIGVDLNLKVTNLM--AGEHLTPEFLKMNPQHTIPTLND---KGFCLWES
GSTδ|G8XWU4                 PCRSVLLAAKAIGVDLNLKVTNLM--AGEHLTPEFLKMNPQHTIPTLND---NGFCLWES
GSTδ|Q9GNE9                 PCRAVQMTAAAVGVELNLKLTNLM--AGEHMKPEFLKINPQHCIPTLVD---NGFALWES
GSTδ|P20432                 PCRSVIMTAKAVGVELNKKLLNLQ--AGEHLKPEFLKINPQHTIPTLVD---NGFALWES
GSTδ|P42860                 PCRSVLMTAKALGIELNKKLLNLQ--AGEHLKPEFLKINPQHTIPTLVD---GDFALWES
GSTδ-like|Q2YFE3            PCRTVSTVAKILNVDMEMKKLDLL--TKEHLNPEFLKVNPFHKVPTFVD--SDGFVVDES
GSTε|Q9GPL9                 PCRAVELTAKALGLELERKLVNLL--AGENLTPEFLKLNPKHTIPVLDD---NGTIISES
GSTε|Q9GPL8                 PCRAVELTAKALGLELEQKTINLL--TGDHLKPEFVKLNPQHTIPVLDD---NGTIITES
GSTσ|P46429                 WARPT-MLLAYGGQEFEDHRVEYE------QWPEFKPNTPFGQMPVLEI---DGKKYAQS
GSTσ|V9Q3X5                 IAEPIRFILAYVGADYEDVRIEFS------DWPPLKPDTPYGTMPVLEV---DGKKLGQS
GSTσ|P41043                 LAEPLRYLFAYGNQEYEDVRVTRD------EWPALKPTMPMGQMPVLEV---DGKRVHQS
GSTσ|Q8MUR9                 LGEPLRFLLSYGNLPFDDVRITRE------EWPALKPTMPMGQMPVLEV---DGKKVHQS
GSTσ|O18598                 LGEPIRFLLSYGEKDFEDYRFQEG------DWPNLKPSMPFGKTPVLEI---DGKQTHQS
GSTσ|V9Q487                 LGEPIRFLLSYGKIEFEDERCERE------KWPSVKESMPFGQMPVLEI---DGKKTWQS
GSTσ|V9Q315                 LGEPIRFLLSYGNMEFEDDRFERE------KWPSIKPSMPFGQVPVLEF---DGKKTHQS
GSTω|Q86D85                 YAQRVHLMLDAKKIPYHAIYINLS-----EKPEWYLEKNPLGKVPALEIPGKEGVTLYES
GSTθ|Q8MUQ1                 PSRALWIFLEKTKLPYEKCLINLG--KGEHLTEEFKAINRFQKVPCITD---SQIKLAES
GSTζ|Q8MUQ5                 CSWRVRIALNLKEIPYDIKPISLIKSGGEQHCNEYREVNPMEQVPALQI---DGHTLIES
                                                                        * :           :*

GST|Accession no.MG255130   RAILSYLADQYGKD---DSLYPKD-AKKRALVDQRLYFDIRTLYHRFGEY----YYPIYF
GSTδ|V9Q2Q3                 HAIVAYLVDRFGKN---DSLYPKD-IQKRSKVNERLCFDISL-FTKVLKF----VVGPLL
GSTδ|V9Q3Y3                 RAISMYLISKYAKD---DSLYPKD-VNKRVLVDQRLFYDQD-LYNRILSV----FLP-KF
GSTδ|V9Q4H6                 RAIAMYLVSRYAKD---DSLYPKE-LQRRVLVDQRLFFDQD-LYNRIMAV----CNP-LF
GSTδ|V9Q328                 VAILQYLADKYGPG---HSLYPRD-PQQRALVNHRLAFNISTYYARIAEY----AVAPIF
GSTδ|V9Q4A2                 RAIIAYLVDNYAKD---DSLYPRD-PKKRATVNRMLFFDAGTLWMNYHIY----QMP-IF
GSTδ|O76483                 RVILAYLVSAYGKD---ENLYPKD-FRSRAIVDQRLHFDLGTLYQRVVDY----YFPTIQ
GSTδ|Q9XZ67                 RAILAYLVNAYGKD---DSLYPKN-PRQRAIVDQRLNFDIGTLFPRYSNL----YFPMLF
GSTδ|Q60GK5                 RAIITYLVNKYAKG---SSLYPED-PKARALVDQRLYFDIGTLYQRFSDY----FYPQVF
GSTδ|Q8N0B3                 RAIMTYLADQYGKD---DSLYPKD-PKKRAKVNQRLYFDMGTLYQSFGDA----YYPHMF
```

```
GSTδ|A9QUN5                RAILSYLADQYGKD---DSLYPKD-PKKRALVDQRLYFDLGTLYQRFGDY----YYPIMF
GSTδ|A7E1B7                RAILSYLADQYGKD---DSLYPKD-PKKRALVDQRLYFDLGTLYQRFGDY----YYPIMF
GSTδ|G8XWV6                RAILSYLADQYGKD---DSLYPKD-PKKRALVDQRLYFDLGTLYQRFGDY----YYPIMF
GSTδ|Q1M0Y4                RAILSYLADQYGKD---DSLYRRD-AKKRALVDQRLYFDIGTLYHRFGEY----YYPIYF
GSTδ|G8XWU4                RAILSYLADQYGKD---DSLYPKD-AKKRALVDQRLYFDIGTLYHRFGEY----YYPIYF
GSTδ|Q9GNE9                RAICTYLAEKYGKD---DKLYPKD-PQKRAVVNQRLYFDMGTLYQRFADY----YYPQIF
GSTδ|P20432                RAIQVYLVEKYGKT---DSLYPKC-PKKRAVINQRLYFDMGTLYQSFANY----YYPQVF
GSTδ|P42860                RAIMVYLVEKYGKN---DSLFPKC-PKKRAVINQRLYFDMGTLYKSFADY----YYPQIF
GSTδ-like|Q2YFE3           RVIAMYLVESRKPD---SFLYPKNDLKKRIQIDRWLHYDINLS-TTISAP----MF-CVF
GSTε|Q9GPL9                HAIMIYLVRKYGQGEGKDALYPTD-IVEQARVNEALHFESGVLFARLRFI----TELVFF
GSTε|Q9GPL8                HAIMIYLVTKYGKD---DSLYPKD-PVKQARVNSALHFESGVLFARMRFN----FERILF
GSTσ|P46429                LAISRYLGRKYGLA-------GND-IEEDFEIDQIVDFVND-IRASAASV----EYEQDA
GSTσ|V9Q3X5                IPICRYLAKQYGLL-------AEN-DWDNAQIDAAVDAIND-LRMSIKNF----YFEENG
GSTσ|P41043                ISMARFLAKTVGLC-------GAT-PWEDLQIDIVVDTIND-FRLKIAVV----SYEPED
GSTσ|Q8MUR9                VAMSRYLANQVGLA-------GAD-DWENLMIDTVVDTVND-FRLKIAVV----SYEPDD
GSTσ|O18598                VAISRYLGKQFGLS-------GKD-DWENLEIDMIVDTISD-FRAAIANY----HYDADE
GSTσ|V9Q487                LAICRYLGKQMGLA-------GAN-DWEDLQIDMAIETVTD-LRLKIANY----WYETDE
GSTσ|V9Q315                IAICRYLGKQLKLA-------GDN-DWEALQIDMAADCLTD-LRMKLGSF----FYESDE
GSTω|Q86D85                LVLSDYIEEAYSAQ--QRKLYPAD-PFSKAQDRILIERFAGS----VIGP----YYRILF
GSTθ|Q8MUQ1                VAIFRYLCREYQVP---DHWYPAD-SRRQALVDEYLEWQHHNTRATCAIYFQYVWLRPRM
GSTζ|Q8MUQ5                VSIMYYLEETRPQ----RPLMPQD-VLKRAKVREICEVIASGV-QPLQNL----IV-LIH
                             :  ::

GST|Accession no.MG255130  AKQAADPEKM----KKLEEAF----EFLNKFLE---SQEFVAGNKLTIADLAIVSSVSTA
GSTδ|V9Q2Q3                RTHEPTEELR----NDCIDGL----ETVERFLS---ASKFIAGDDLTVADYCYYCTITFV
GSTδ|V9Q3Y3                FGKQTDPSSI----EKVNEGL----ETLNRMLD---GKQWLAGDNITLADYAIANTLSAL
GSTδ|V9Q4H6                YGKTVAESDA----DRMKDGL----DTLSRMLD---GKQWLAGDHVTLADYAVAVSLATL
GSTδ|V9Q328                FDYKRTPEGL----NKLKIAL----NVLNTILERQ-GTKFAAGEHMTLADLSLVAATMCL
GSTδ|V9Q4A2                YGQPGDPEKQ----KKMLEAF----ALFDKLLE---GKEWAAGSHVTIADYALAVTATSA
GSTδ|O76483                LGAHLDQTKK----AKLAEAL----GWFEAMLK---QYQWSAANHFTIADIALCVTVSQI
GSTδ|Q9XZ67                RGDEYNQENA----DKLNEAL----GWLNIFLE---KSAFVAGDNLTIADISIIVTITNL
GSTδ|Q60GK5                AGAPADKAKN----EKVQEAL----QLLDKFLE---GQKYVAGPNLTVADLSLIASVSSL
GSTδ|Q8N0B3                GGAPLDEDKK----KKLGDAL----VFLDGFLE---KSAFVAGEDLTLADLAIVASISTI
GSTδ|A9QUN5                AKASPDAEKM----KKLEEAY----QFLDKFLE---GQKFVAGNSLTIADIATIASVSTA
GSTδ|A7E1B7                AKASPDAEKM----KKLEEAY----QFLDKFLE---GQKFVAGNSLTIADIATIASVSTA
GSTδ|G8XWV6                AKASPDAEKM----KKLEEAY----QFLDKFLE---GQKFVAGNSLTIADIATIASVSTA
GSTδ|Q1M0Y4                AKQAADPEKM----KKLEEAF----EFLNKFLE---SQEFVAGNKLTIADLAIVSSVSTA
GSTδ|G8XWU4                AKQAADPEKM----KKLEEAF----EFLNKFLE---SQEFVAGNKLTIADLAIVSSVSTA
GSTδ|Q9GNE9                AKQPANAENE----KKMKDAV----DFLNTFLD---GHKYVAGDSLTIADLTVLATVSTY
GSTδ|P20432                AKAPADPEAF----KKIEAAF----EFLNTFLE---GQDYAAGDSLTVADIALVATVSTF
GSTδ|P42860                AKAPADPELY----KKMEAAF----DFLNTFLE---GHQYVAGDSLTVADLALLASVSTF
GSTδ-like|Q2YFE3           RGHQVQDYQV----EQGKETL----KTLDGVMQSFEGKFLTGADQFTLADIAMYFSLNTM
GSTε|Q9GPL9                ARKPEIPEDR---IEYVRTAY----RLLEDSL----QSDYVAGSRMTIADLSCISSVASM
GSTε|Q9GPL8                FGKSDIPEDR---VEYVQKSY----ELLEDTL----VDDFVAGPTMTIADFSCISTISSI
GSTσ|P46429                ANKEVK------HEENMKNKYPFQLNKLSEIIT--KNNGFLALGRLTWADFVFVGMFDYL
GSTσ|V9Q3X5                STKNNM------KEKLMKEAVPFYYKKLEEMVK--NNGGYLAGGKLTWGDLYLTTMLELF
GSTσ|P41043                EIKEKK------LVTLNAEVIPFYLEKLEQTVK--DNDGHLALGKLTWADVYFAGITDYM
GSTσ|Q8MUR9                EIKEKK------LVTLNNEVIPFYLEKLDDIAR--DNNGYLANSKLSWADIYFTAILDYL
GSTσ|O18598                NSKQKK------WDPLKKETIPYYTKKFDEVVK--ANGGYLAAGKLTWADFYFVAILDYL
GSTσ|V9Q487                AQKEKK------KEPLFNETLPFLLPRLDNLVK--ENGGYLANGKLSWADLYFIAVLDYL
GSTσ|V9Q315                AVKEKK------KEPIIKEFLPFFLPRLDNLVK--ENGGYLANGKLSWADFYFAGILDYV
GSTω|Q86D85                AADGIPPGAI----TEFGAGL----DIFEKELKA-RGTPYFGGDKPGMIDYMIWPWCERV
GSTθ|Q8MUQ1                FGTKVDPKQAEKYRGQMEGTL----DFIEREYLG-SGARFIAGDEITVADLLAACEIEQP
GSTζ|Q8MUQ5                VGEEKKKEWA---QHWITRGF----RAIEKLLST-SAGKFCVGDEITLADCCLVPQVFNA
                                                      ..                    *

GST|Accession no.MG255130  --------DIMGF-DV-SKYSNVAKWFEKCKKIVPGYE--ELNHSGCLKFKEMCDNLAKK
GSTδ|V9Q2Q3                -----DIPQKGVI-DL-KKYKNIQRWMDLIHKTCPLFT--KYDQIAEKAFQIYLEDGPY-
GSTδ|V9Q3Y3                -----EFAPEFGI-DP-TKQPNIKQWLPRVESSSPKYG--ESL----KEFREALKKMAQK
GSTδ|V9Q4H6                -----EYNPEFAI-DA-AKHPNITQWISRLEESSNTYR--EHI----ELFFETVRKATEE
GSTδ|V9Q328                --------EAVEF-DL-TPWARVQRWYADFKQAAPRLW--AVAEPAMLELRAFSNSPPDL
GSTδ|V9Q4A2                --------QWSGV-DL-KVYDNVTRWLERTKEGITCFD--EINNSSFAALKELLQKLKEK
GSTδ|O76483                --------EAFQF-DL-HPYPRVRAWLQKCKDELQGHGYKEINETGAETLAGLFRSKLKQ
GSTδ|Q9XZ67                --------DAFKF-DF-SEHENLTKWFERTKKALEPYDWEDIDETGAQMLADFLKREH--
GSTδ|Q60GK5                --------EASDI-DF-KKYANVKRWYETVKSTAPGYQ--EANEKGLEAFKGLVNSMLKK
GSTδ|Q8N0B3                --------EAVEY-DL-SPYKNINSWYSKVKAAAPGYK--EANEEGAKGFGQMFKAMTGK
GSTδ|A9QUN5                --------AILGF-DI-TRYPNVNKWFENAKKVIPGYD--ELNHSGCLEFRKMWDNLTQK
GSTδ|A7E1B7                --------AILGF-DI-TRYPNVNKWFENAKKVIPGYD--ELNHSGCLEFKKMWDNLTQK
GSTδ|G8XWV6                --------AILGF-DI-TRYPNVNKWFENAKKVIPGYE--ELNHSGCLEFKKMWDKLTQK
GSTδ|Q1M0Y4                --------DIMGF-DV-SKYSNVAKWFEKCKKIVPGYE--ELNHSGCLKFKEMCDNLAKK
GSTδ|G8XWU4                --------DIMGF-DV-SKYSNVAKWFEKCKKIVPGYE--ELNHSGCLKFKEMCDNLAKK
GSTδ|Q9GNE9                --------DVAGF-EL-AKYPHVAAWYERTRKEAPGAA--I-NEAGIEEFRKYFEK----
GSTδ|P20432                --------EVAKF-EI-SKYANVNRWYENAKKVTPGWE--E-NWAGCLEFKKYFE-----
GSTδ|P42860                --------EVAGF-DF-SKYANVAKWYANAKTVAPGFD--E-NWEGCLEFKKFFN-----
GSTδ-like|Q2YFE3           -----EVYP-KYF-KF-DDYPNLKSWYHRVAEALKQYDTEGTIPKAIETMKQFIQQRAAE
GSTε|Q9GPL9                -----V--GFIPM-ER-SEFPRVHGWIERMKQ-LPYYE--EINGAGATELAEFIVDMLAK
GSTε|Q9GPL8                -----M--GVVPL-EQ-SKHPRIYAWIDRLKQ-LPYYE--EANGGGTDLGKFVLAKKEE
```

```
GSTσ|P46429                 -------KKMLRMPDLEEQYPIFKKPIETVLSNPKLKAYLDSAPKKEF------------
GSTσ|V9Q3X5                 -------DHDLEC-EITKDYPHLATLRNKVVNIPSIKTWIEKRPKSER------------
GSTσ|P41043                 -------NYMVKR-DLLEPYPALRGVVDAVNALEPIKAWIEKRPVTEV------------
GSTσ|Q8MUR9                 -------NYMTKS-DLVANHPNLQRVVDNVTSIESIRSWIDKRPKTEI------------
GSTσ|O18598                 -------NHMAKE-DLVANQPNLKALREKVLGLPAIKAWVAKRPPTDL------------
GSTσ|V9Q487                 -------NTMVGF-DLTKDYPNLAELKNKVLEIPAIKEWVDKRPKTDL------------
GSTσ|V9Q315                 -------NHMAGF-DITKDYANLAALKNKVLELPAIKEWIAKRPVSEV------------
GSTω|Q86D85                 DLLKFALGDKYEL-DK-ERFGKLLQWRELMEKDDAVKQ----SFISTEDHTKFLQSRKNG
GSTθ|Q8MUQ1                 --------RMAGY-DPCEGRPNLTQWMARVRESTNPYY--DQAHK---LVNKFAQDTASK
GSTζ|Q8MUQ5                 --------RRFHV-DL-RPYPIILRID-RELEGHPAFR--AAHPS---------------
                                          .       .

                                             % identity (compared to MG255130)
GST|Accession no.MG255130   --------------   100.0
GSTδ|V9Q2Q3                 --------------    32.9
GSTδ|V9Q3Y3                 --------------    43.8
GSTδ|V9Q4H6                 EKKNAQQK------    33.6
GSTδ|V9Q328                 SALRHPYHPVRASK    34.6
GSTδ|V9Q4A2                 LAEMSVD------K    42.7
GSTδ|O76483                 --------------    46.0
GSTδ|Q9XZ67                 --------------    48.4
GSTδ|Q60GK5                 --------------    55.6
GSTδ|Q8N0B3                 --------------    60.6
GSTδ|A9QUN5                 --------------    81.5
GSTδ|A7E1B7                 --------------    82.4
GSTδ|G8XWV6                 --------------    81.9
GSTδ|Q1M0Y4                 --------------    98.1
GSTδ|G8XWU4                 --------------    99.5
GSTδ|Q9GNE9                 --------------    58.3
GSTδ|P20432                 --------------    61.6
GSTδ|P42860                 --------------    61.1
GSTδ-like|Q2YFE3            AEKH----------    31.2
GSTε|Q9GPL9                 NAKL----------    39.5
GSTε|Q9GPL8                 NAKA----------    39.6
GSTσ|P46429                 --------------    22.7
GSTσ|V9Q3X5                 --------------    21.2
GSTσ|P41043                 --------------    11.0
GSTσ|Q8MUR9                 --------------    17.5
GSTσ|O18598                 --------------    16.8
GSTσ|V9Q487                 --------------    23.8
GSTσ|V9Q315                 --------------    21.7
GSTω|Q86D85                 ENNYDILA------    18.8
GSTθ|Q8MUQ1                 AKL-----------    26.5
GSTζ|Q8MUQ5                 ---NQPDCPPEAAK    23.3
```

**Supplementary Figure 2. Alignment (Clustal Omega Multiple Sequence Alignment) and percent identity of amino acid sequence of *P. americana* rGST of this study (accession number MG255130) with GSTs of other insects in Figure 1.**

```
          1         11        21        31        41        51        60
          |         |         |         |         |         |        |
          MTIDFYYLPGSAPCRSVLLAAKAIGVDLNLKVTNLMAGEHLTPEFLKMNPQHTIPTLNDN
            <-β1>    <----α1----->  <-β2>          <-α2->       <β3>
BCPred    ...............................................MNPQHTIPTLNDN
AAP       ................................................NPQHTIPTLNDN
FBCPred   MTIDFYYLPGSAPCRSVLLA.............................PQHTIPTLNDN
Pep1      MTIDFYYLPGSAPCRSVLLA----------------------------------------
Pep2      -----------------------------------------------MNPQHTIPTLNDN
Pep3      ------------------------------------------------------------
Pep4      ------------------------------------------------------------
Pep5      ------------------------------------------------------------
Pep6      ------------------------------------------------------------

          61        71        81        91        101       111       120
          |         |         |         |         |         |        |
          GFCLWESRAILSYLADQYGKDDSLYPKDAKKRALVDQRLYFDIRTLYHRFGEYYYPIYFA
           <β4> <----α3---->          <-----α4------>   <----α5----->
BCPred    GFCLWES................................................PIYFA
AAP       GFCLWESR........QYGKDDSLYPKDAKKRALVD........................
FBCPred   GFCLWESRA..SYLADQYGKDDSLYPKDAKK..........................YFA
Pep1      ------------------------------------------------------------
Pep2      GFCLWESRA---------------------------------------------------
Pep3      GFCLWESRAILSYLADQYGK----------------------------------------
Pep4      -----------SYLADQYGKDDSLYPKDAKKRALVD------------------------
Pep5      -------------------------------------------------------PIYFA
Pep6      ------------------------------------------------------------

          121       131       141       151       161       171       180
          |         |         |         |         |         |        |
          KQAADPEKMKKLEEAFEFLNKFLESQEFVAGNKLTIADLAIVSSVSTADIMGFDVSKYSN
               <-------α6------->            <------α7------>       <-
BCPred    KQAADPEKMKKLEEA.............................................
AAP       ............................................................
fbcpred   KQAADPEKMKKLEEAFE...........................................
Pep1      ------------------------------------------------------------
Pep2      ------------------------------------------------------------
Pep3      ------------------------------------------------------------
Pep4      ------------------------------------------------------------
Pep5      KQAADPEKMKKLEEAFE-------------------------------------------
Pep6      ------------------------------------------------------------

          181       191       201       211
          |         |         |         |
          VAKWFEKCKKIVPGYEELNHSGCLKFKEMCDNLAKK 216
          ---α8----->       <-------α9------>
BCPred    ....................................
AAP       ......KCKKIVPGYEELNHSGCLKF..........
FBCPred   ....................................
Pep1      ------------------------------------
Pep2      ------------------------------------
Pep3      ------------------------------------
Pep4      ------------------------------------
Pep5      ------------------------------------
Pep6      ------KCKKIVPGYEELNHSGCLKF----------
```

**GST-dv2 B-cell epitope prediction**

**BCPREDS Server 1.0**

------------------------------------

**Submitted sequence:** 216 amino acids
**Epitope length:** 20 amino acids
**Classifier Specificty:** 85%
**Prediction method:** bcpred
**Use overlap filter:** yes

------------------------------------

**BCPred Predictions**

| Position | Epitope | Score |
|---|---|---|
| 48 | MNPQHTIPTLNDNGFCLWES | 0.945 |
| 116 | PIYFAKQAADPEKMKKLEEA | 0.932 |

```
1         11        21        31        41        51        60
|         |         |         |         |         |         |
MTIDFYYLPGSAPCRSVLLAAKAIGVDLNLKVTNLMAGEHLTPEFLKMNPQHTIPTLNDN 60
...............................................EEEEEEEEEEEEE
GFCLWESRAILSYLADQYGKDDSLYPKDAKKRALVDQRLYFDIRTLYHRFGEYYYPIYFA 120
EEEEEEE................................................EEEEE
KQAADPEKMKKLEEAFEFLNKFLESQEFVAGNKLTIADLAIVSSVSTADIMGFDVSKYSN 180
EEEEEEEEEEEEEEE.............................................
VAKWFEKCKKIVPGYEELNHSGCLKFKEMCDNLAKK 216
....................................
```

**BCPREDS Server 1.0**

------------------------------------

**Submitted sequence:** 216 amino acids
**Epitope length:** 20 amino acids
**Classifier Specificty:** 85%
**Prediction method:** aap
**Use overlap filter:** yes

------------------------------------

**AAP Predictions**

| Position | Epitope | Score |
|---|---|---|
| 187 | KCKKIVPGYEELNHSGCLKF | 1 |
| 49 | NPQHTIPTLNDNGFCLWESR | 1 |
| 77 | QYGKDDSLYPKDAKKRALVD | 1 |

```
1         11        21        31        41        51        60
|         |         |         |         |         |         |
MTIDFYYLPGSAPCRSVLLAAKAIGVDLNLKVTNLMAGEHLTPEFLKMNPQHTIPTLNDN 60
................................................EEEEEEEEEEEE
GFCLWESRAILSYLADQYGKDDSLYPKDAKKRALVDQRLYFDIRTLYHRFGEYYYPIYFA 120
EEEEEEEE........EEEEEEEEEEEEEEEEEEEE........................
KQAADPEKMKKLEEAFEFLNKFLESQEFVAGNKLTIADLAIVSSVSTADIMGFDVSKYSN 180
............................................................
VAKWFEKCKKIVPGYEELNHSGCLKFKEMCDNLAKK 216
......EEEEEEEEEEEEEEEEEEEE..........
```

**BCPREDS Server 1.0**

```
------------------------------------
Submitted sequence: 216 amino acids
Epitope length: 20 amino acids
Classifier Specificty: 85%
Prediction method: fbcpred
Use overlap filter: yes
------------------------------------
FBCPred Predictions
```

| Position | Epitope | Score |
|---|---|---|
| 118 | YFAKQAADPEKMKKLEEAFE | 1 |
| 72 | SYLADQYGKDDSLYPKDAKK | 0.999 |
| 1 | MTIDFYYLPGSAPCRSVLLA | 0.98 |
| 50 | PQHTIPTLNDNGFCLWESRA | 0.975 |

```
1         11        21        31        41        51       60
|         |         |         |         |         |        |
MTIDFYYLPGSAPCRSVLLAAKAIGVDLNLKVTNLMAGEHLTPEFLKMNPQHTIPTLNDN 60
EEEEEEEEEEEEEEEEEEEE.............................EEEEEEEEEEE
GFCLWESRAILSYLADQYGKDDSLYPKDAKKRALVDQRLYFDIRTLYHRFGEYYYPIYFA 120
EEEEEEEEE..EEEEEEEEEEEEEEEEEEEE..........................EEE
KQAADPEKMKKLEEAFEFLNKFLESQEFVAGNKLTIADLAIVSSVSTADIMGFDVSKYSN 180
EEEEEEEEEEEEEEEEE...........................................
VAKWFEKCKKIVPGYEELNHSGCLKFKEMCDNLAKK 216
....................................
```

**Supplementary Figure 3. Results of the *P. americana*-GST B-cell epitope prediction by using three different methods of the BepiPred 1.0 server.** There were 2, 3, and 4 epitopes derived from the BCPred, AAP, and FBCPred prediction. These epitopes were aligned with the *P. americana*-GST sequence and they were matched to five different regions of the GST linear sequence. Peptides of these regions were synthesized. Because the regions 2 and the next were closed to each; therefore, a peptide that overlapped the two regions was synthesized. All six peptides were tested for binding to IgG and IgE in *P. americana* allergic patients' sera to determine their B-cell epitopes and allergenic epitopes.
